# Mucocele of the dorsal surface of the tongue: A case report

**DOI:** 10.4317/jced.54497

**Published:** 2018-05-01

**Authors:** Savvas Titsinides, Demos Kalyvas, Konstantinos Tosios

**Affiliations:** 1 Department of Oral Medicine and Pathology, Dental School, University of Athens, Greece; 2 Department of Oral and Maxillofacial Surgery, Dental School, University of Athens, Greece

## Abstract

Mucoceles represent one of the most common lesions of the oral cavity, developing as a result of saliva accumulation. The most frequent affected area is the lower lip, followed by floor of mouth, ventral tongue and buccal mucosa. Despite numerous reports of mucoceles originating on the ventral surface of the tongue, only scarce cases of such a lesion identified on the dorsal tongue surface have been described. In this report a mucocele developed on the dorsal tongue of a 74-year-old woman is described. Additionally a review of previously published mucoceles of the dorsal surface of the tongue is provided and discussed. A 74-year-old female patient was referred for a painless swelling on the dorsal surface of the tongue of 1 month duration. Possible clinical diagnosis included granular cell tumor and lingual thyroid gland. Proper blood testing for TSH, T3 and T4 as well as ultrasonography were requested, found to be within normal limits. An excisional biopsy was performed and tumor was removed with no intra-operative complications. Histopathological examination was consistent with a mucocele, exhibiting an amorphous material surrounded by granular connective tissue without epithelial lining on the periphery. Patient was examined on regular follow-up basis, with no signs of recurrence for the last 1 year. Mucoceles of the dorsal tongue surface represent rare clinical entities, necessitating the need for further case reports to be published in order to widen our understanding of their clinical features.

** Key words:**Mucocele, oral cavity, tongue, minor salivary glands, oral and maxilloafcial pathology.

## Introduction

Mucoceles develop in the oral cavity as a result of saliva accumulation, causing swelling of the affected area. These entities represent one of the most common lesions of the oral mucosa while they are likely to grow at a lesser frequency in other parts of the human body such as the nasal cavity and maxillary sinus (1).

They are mostly subdivided into two categories: a. Mucus extravasation type; rupture of salivary glands’ duct as a result of injury can lead to leakage and concentration of saliva in the surrounding tissues. In this way a cavity is formed within the soft tissues, not surrounded by epithelial wall lining, developing a pseudocyst, and b. Mucus retention type; obstruction of the salivary duct leading to a true cyst covered by epithelium. About 90% of such lesions are classified as extravasation mucosal cysts while only 10% as mucous retention cysts (2).

The incidence of oral mucoceles is difficult to be estimated due to the fact that a large percentage of these lesions are not referred for histopathological examination.

It should be highlighted that mucoceles may develop at any age. Nevertheless, among the studies, a higher incidence of both the 2nd and the 3rd decade of life is reported with no sex predilection. Importantly, among the 2 subtypes, the mucous extravasation cyst occurs at younger ages compared to those from retention (3).

Mucous cysts, irrespective of their etiology, appear as soft, asymptomatic swellings with a color ranging from deep blue to the color of normal mucosa. A common finding is the relatively periodic disappearance and recurrence of the lesion as the cystic cavity is subjected to rupture and re-aggregation of saliva. Often after rupture mucoceles leave superficial painful ulcerations that heal within days (4).

The most frequent region of development is the lower lip, followed by floor of mouth, ventral tongue and buccal mucosa. It is expected that mucoceles are relatively not frequent in the upper lip, compared to the lower lip since the latter is more easily to be injured. Regarding the tongue, mucoceles are also observed on the anterior ventral surface of the tongue where the Blandin-Nuhn sero-mucous salivary glands exist. Also, in proximity to the lingual tonsils, the glands of Weber, exclusively mucous glands are identified. Finally in the periphery of circumvallate papillae and the base of the clefts between the foliate papillae the serous salivary glands of von Ebner are located (5).

Differential diagnosis of dorsal tongue swellings include lingual ectopic thyroid, thyroglossal duct cyst, dermoid/epidermoid cysts as well as granular cell tumor, lymphangiomas and hemangiomas. However, using ultrasonography or MRI as diagnostic aids, lymphangiomas, hemangiomas as well as dermoid/epidermoid cysts have a relatively distinct appearance, offering a way to differentiate them. Thyroglossal duct cysts are located at a relatively posterior area, usually at the tongue base, from the foramen cecum to the thyroid gland. Lingual ectopic thyroid gland is a rare developmental anomaly caused by lack of descent of all or part of the embryonic thyroid gland. Diagnostic exams for such a lesion include hormonal tests – TSH, T3 and T4 – as well as ultrasonography to evaluate its anatomic presence in the lower neck. Finally, regarding granular cell tumor its clinical aspects, especially elasticity on palpation is quite helpful to discriminate it from relatively soft lesions like a mucocele.

Despite numerous reports of mucoceles originating on the ventral surface of the tongue, there have been only scarce reports of such a cyst forming on the dorsal surface. This article describes a mucous extravasation cyst developed on the dorsal tongue of a 74-year-old woman. Additionally a review of previously published mucoceles of the dorsal surface of the tongue is provided and discussed.

## Case Report

**Figure 1:**
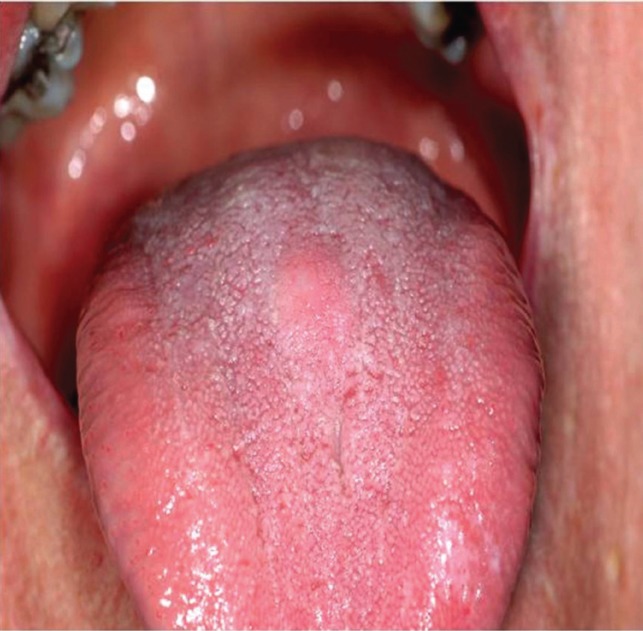
Macroscopic appearance of the lesion on the middle third of tongue’s dorsal surface.

**Figure 2:**
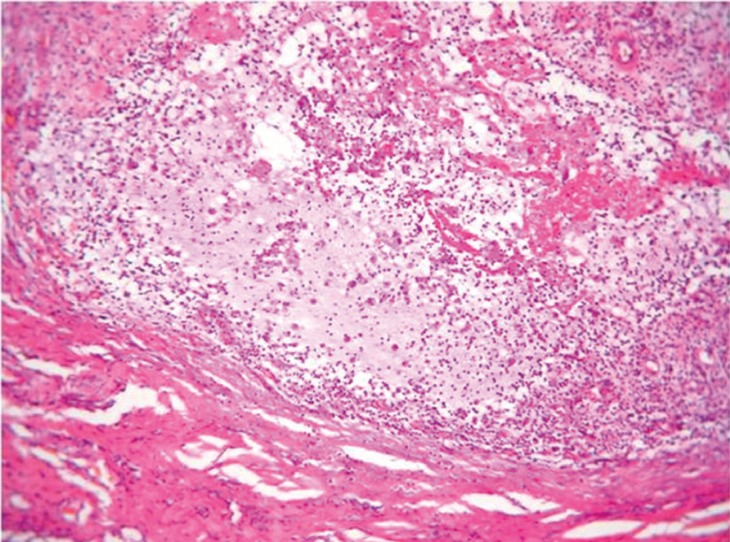
Microphotograph exhibiting a cystic cavity with absence of epithelial lining, surrounded by granulation tissue. (Haematoxylin – eosin stain, original magnification x 100).

**Figure 3:**
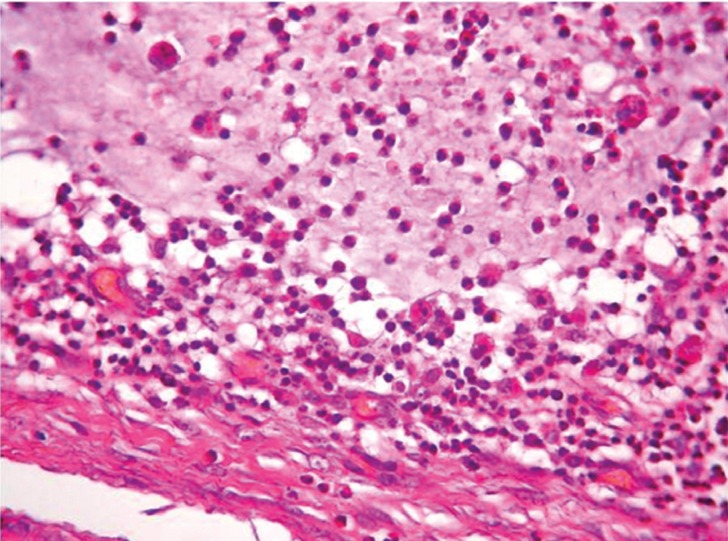
In the lesions’ periphery, fibrous connective tissue and numerous foamy histiocytes are identified. (Haematoxylin – eosin stain, original magnification x 400).

A 74-year-old female patient was referred to the Department of Oral and Maxillofacial Surgery complaining for a painless swelling on the dorsal surface of the tongue of 1 month duration. Her general health status was satisfactory, presenting no systemic diseases. On clinical examination a relatively soft mass of about 1.5cm in maximum diameter, covered by normal epithelium was identified on the centre of middle third of the dorsal surface of the tongue (Fig. 1). Possible clinical diagnosis included granular cell tumor and lingual thyroid gland. Proper blood testing for TSH, T3 and T4 as well as thyroid ultrasonography were requested, found to be within normal limits. An excisional biopsy was performed and tumor was removed with no intra-operative complications. Histopathological examination was consistent with a mucocele, exhibiting an amorphous material surrounded by granular connective tissue without epithelial lining on the periphery (Fig. 2). There were numerous foamy histiocytes and some monomorphonuclear leucocytes accompanying the mucus in the cavity (Fig. 3). Patient was examined on regular follow-up basis, exhibiting no signs of recurrence for the last 1 year.

## Discussion

As previously described, there are 3 distinct sets of oral minor salivary glands in the tongue: the glands of Blandin-Nuhn, the glands of Weber and the glands of von Ebner. The glands of Blandin-Nuhn are composed of sero-mucous acini, located in the anterior ventral surface of the tongue. The glands of Weber are exclusively mucous glands, located close to the lingual tonsils. Finally, the glands of von Ebner are serous salivary glands, located at the base of the grooves that surround the circumvallate papillae and the base of the clefts between the foliate papillae.

Focusing in the tongue, as an anatomic structure, mucoceles develop usually in the ventral surface (mucoceles of Blandin-Nuhn glands) (6). Normally, according to anatomic studies, there are no minor salivary glands on the dorsal surface of the tongue. However, to the best of our knowledge scarce case reports have been published, describing mucoceles of the dorsum of the tongue. As an effort to collect all these rare cases, we performed a comprehensive search of the English language literature using PubMed/MEDLINE electronic database; all published mucoceles of the dorsum of the tongue were reviewed, and their demographic / clinicopathologic data are presented in  Table 1.

**Table 1:**
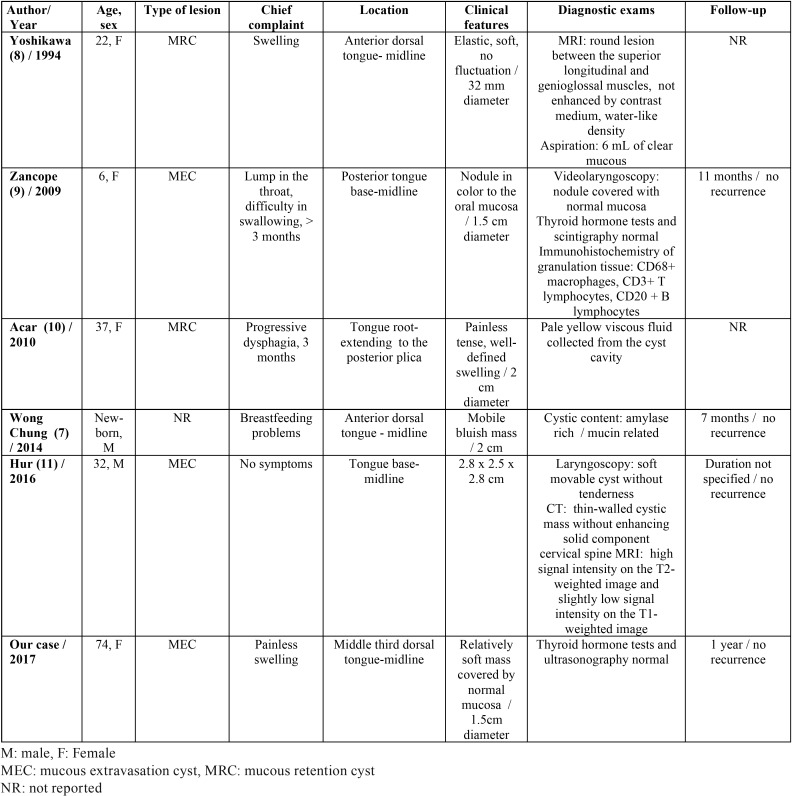
Data summary of mucoceles developed in the dorsal surface of the tongue published in the English language literature.

According to collected data, a wide age range of patients ranging from birth to 74 years old was recorded.

Mucous cysts were identified in the anterior as well as in the posterior area of the dorsal surface of the tongue, exhibiting no predilection.

Regarding their aetiology, we can speculate that lesions of the anterior dorsal tongue may develop from Blandin-Nuhn glands while those of the posterior area may derive from Weber and von Ebner salivary glands. Wong Chung *et al.* suggested that a mucous cyst identified in a newborn most likely originated from an anterior lingual gland which drained through a small, closed ended, duct into the dorsal tongue (7). In our case we may also hypothesize that a minor salivary gland tissue was entrapped between the first or second branchial arches when they fused, further forming the mucocele due to possible irritating factors e.g. trauma.

According to the histopathological criteria there were 3 mucous extravasation cysts, 2 mucous retention cysts while in 1 case there were no data available specifying its type.

Mucoceles on the posterior surface / base of the tongue are possibly more challenging to diagnose, necessitating special diagnostic examinations e.g. laryngoscopy while those in the anterior surface usually require a thorough clinical examination with no further exams. Also those lesions in the tongue base were found to provoke more severe symptoms including dysphagia and difficulty in swallowing while they were all removed under general anaesthesia.

Although the limited number of intraoral mucoceles developed in the dorsal surface of the tongue precludes definitive conclusions, abovementioned discussion includes author’s remarks / observations from the collected cases.

Mucoceles of the dorsal tongue surface represent rare clinical entities, due to the fact that there are no salivary glands located on this area. It seems that they develop due to pathophysiologic mechanisms, that further require to be discovered. Due to their scarce description the need for future case reports to be published is imperative in order to widen our understanding of their clinical features.
